# Editorial: Target-Triggered Nanoparticles for Tumor Diagnosis and Therapy

**DOI:** 10.3389/fchem.2021.657782

**Published:** 2021-04-12

**Authors:** Yi Hou, Xiaoling Wei, Yunfeng Yan, Jing Hao

**Affiliations:** ^1^ College of Life Science and Technology, Beijing University of Chemical Technology, Beijing, China; ^2^ Shanghai Institute of Microsystem and Information Technology, Chinese Academy of Sciences, Shanghai, China; ^3^ University of Chinese Academy of Sciences, Beijing, China; ^4^ College of Biotechnology and Bioengineering, Zhejiang University of Technology, Hangzhou, China; ^5^ Department of Chemistry, George Fox University, Newberg, OR, United States

**Keywords:** cancer theranostics, Molecular imaging, drug delivery, precision medicine, activatable nanomedicine

Cancer is one of the major diseases which seriously jeopardizes the human health and life; however, there remains a lack of effective methods for early diagnosis, metastasis warning, clinical efficacy prediction, and effective treatment. Precision medicine has been heralded to bring more strategies to improve the diagnostics sensitivity and treatment efficacy of malignant tumor (Omidi, 2011; Kelkar and Reineke, 2011).

The risk of cancer is related to its malignant behaviors, including proliferation, invasion, and metastasis, which are closely associated with variations in physiological parameters, such as hypoxia, low extracellular pH, enzyme, and reducing conditions (Ma et al., 2018). These tumor-associated physiological parameters are not merely hallmarks for distinct cancer, they could also serve as targets for constructing tumor-specific medicines, especially as trigger to activate the imaging probes or antitumor drugs (Gao et al., 2017; Janib et al., 2010). In the first 2 decades of this century, many efforts have been made to develop novel theranostic reagents, and fruitful achievements have been reported (Zhang et al., 2019; Gu et al., 2019). Among these candidates, the functional nanoparticle-based drugs are considered to be promising tumor theranostic reagents because their large specific surface area offers a big room to modified functional moieties, which could respond to the stimulus in tumor microenvironments (Gao et al., 2016 ). The aim of this topic was to report the latest achievements in the designs of “smart” nanomedicine responding to the tumor microenvironment-related features, in order to improve the tumor imaging diagnosis and therapy.

Monitoring the tumor-associated microenvironmental physiological parameters and clarifying their relationship are critical both for tumor diagnostics and therapeutic administrations. Therefore, Hou et al. have reviewed the recent achievements in target-triggered nanoprobes for tumor theranostics, including the preparation strategies, response mechanisms, and theranostic applications of the state-of-the art activable theranostic nanoprobe. The target-triggered tumor theranostic nanoprobes have successfully associated various imaging modalities with distinct treatment approaches of tumor, including chemotherapy, chemodynamic therapy, gene therapy, immunotherapy, and physical therapy. Owing to the integration of both diagnosis and treatment of cancer, these nanoprobes are obviously superior to the conventional probes. Thus, the target-triggered nanoprobes exhibit powerful abilities to not only monitor and trace the *in vivo* behavior of themselves but also give an immediate feedback on the treatment outcome without time consumption, thereby evaluating the prognosis in real time.

The target-triggered nanoparticles have also been considered as promising delivery systems, which could realize the targeted delivery of antitumor drugs, reduced systemic toxicity, and improved therapeutic effect. Various stimuli-responsive carriers have been developed for tumor therapeutics; among them, the enzyme-activated nanoparticles are considered as one of the most promising smart stimulus-responsive nanocarriers because the changes in the expression of specific enzymes, such as proteases, phosphatases, and glycosidases, have been observed in tumor or inflammatory regions, which can be exploited to achieve targeted accumulation of drugs at the desired biological location *via* enzyme-mediated drug release. Li et al. have reviewed the significant progress in the enzyme-responsive nanoparticles for antitumor drug delivery in the past years. They have summarized the general mechanism for controlling drug release from the enzyme-responsive carriers and highlighted the state of the art in drug delivery and disease diagnosis systems based on enzyme-responsive nanoparticles, which are organized based on different installation sites of specific enzyme bioactive functionalities on nanoparticles.

Besides enzyme, the concentrated redox species, such as glutathione (GSH), is another common phenomenon in a majority of solid tumors. Pang et al. have developed integrated nanocarriers containing FA ligands and ditelluride bonds for active tumor-targeting and GSH-responsive drug release. They synthesized a folic acid (FA)-modified PEGylated polycaprolactone (PEG-PCL) containing ditelluride linkage through a facile coupling reaction. And then, the hydrophobic doxorubicin (DOX) can be encapsulated into the polymeric micelles (F-TeNP_DOX_) *via* self-assembly in aqueous solution. The nanomedicine could target tumor cells, and be efficiently internalized into the cells through FA-mediated endocytosis and achieve sufficient “active-drug” content after redox-responsive micelle dissociation.

Wang et al. introduced a redox-triggered dual-targeted liposome, CEP-LP@S/D, capable of co-delivering DOX and salinomycin (Sal) for the synergistic treatment of liver cancer. They designed CD133, and EpCAM dual-targeted Y-shaped peptide ligand, CEP, and decorated the surface of this liposome with the peptide, in order to improve both recognition and binding to cancer stem cell (CSC) subpopulations, which is believed to be associated with high chemoresistance and recurrence rates in hepatocellular carcinoma (HCC). Moreover, they endowed the CEP-LP@S/D with GSH-responsive properties to initiate anticancer drug release. The *in vitro* and *in vivo* studies indicate that their GSH-responsive co-delivery system could not only effectively enhance CSC targeting but also eliminate the non-CSC faction, thereby exhibiting high antitumor efficacy.

The external stimulus, such as photothermal effect, is considered to be employed to design precise nanomedicines. Wang et al. have developed a dual-functional micellar drug delivery system based on thermosensitive poly(N-isopropylacrylamide) (PNIPAM), poly(d,l-lactide)-poly (ethylene glycol) (PLA-PEG), and gold nanorods (GNRs). The heat generated by the GNRs under near-infrared light irradiation induces shrinking of the PNIPAM, which in turn will promote drug release and achievement of higher local drug concentration at the tumor site, leading to potent *in vivo* tumor inhibitory activity.

Photodynamic therapy (PDT) has become an alternative approach to treat tumors through reactive oxygen species (ROS) produced by the activated photosensitizers (PS). Liu et al. have reviewed the nanoparticles which could respond to tumor-associated microenvironments, mainly including pH, redox species, enzymes, and hypoxia, and highlight the applications of these smart nanomaterials as targeted delivery carriers of PS in photodynamic anticancer therapy, to further boost the development of PDT in tumor therapy.

This research topic introduces the state of the art in target-triggered tumor theranostic nanoprobes. The fruitful achievements have been gained, which exhibit great potential not only in tumor theranostics but also in other serious diseases such as Alzheimer’s disease and stroke.
